# Supplementary material for: Effect of Beetroot Powder Incorporation on Functional Properties and Shelf Life of Biscuits
Source: Foods. 2023 Jan 9;12(2):322. doi: 10.3390/foods12020322 (PMC9858528; doi:10.3390/foods12020322)
Supplement: Supplementary file 1 [file foods-12-00322-s001.zip › foods-2104697-supplementary.pdf]

Supplementary Materials

Effect of Beetroot Powder Incorporation on Functional Properties and Shelf Life of Biscuits

Jasmina Mitrevski [1,2], Nebojša Đ. Pantelić [1*], Margarita S. Dodevska [3], Jovana S. Kojić [4], Jelena J. Vulić [5], Snežana Zlatanović [6], Stanislava Gorjanović [6], Jovanka Laličić-Petronijević [1], Sonja Marjanović [7] and Vesna V. Antić [1]

* Correspondence: pantelic@agrif.bg.ac.rs

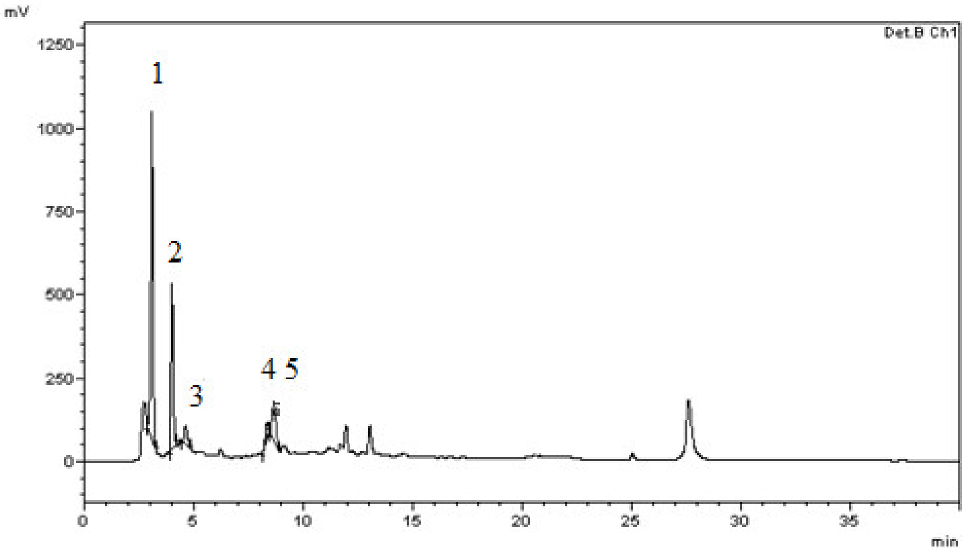

Figure S1. HPLC chromatograms of phenolics in beetroot power (start of storage) on 280 nm: 1 - gallic acid; 2 - protocatechuic acid; 3 - catechin; 4 - epicatechin; 5 - vanillic acid.

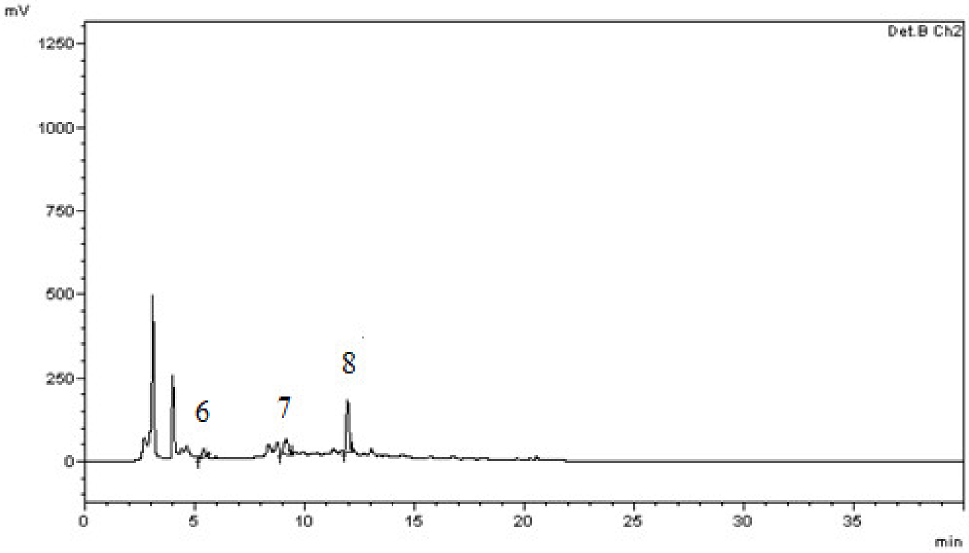

Figure S2. HPLC chromatograms of phenolics in beetroot power (start of storage) on 320 nm: 6 - chlorogeni acid; 7 - *p*-coumaric acid; 8 - caffeic acid.

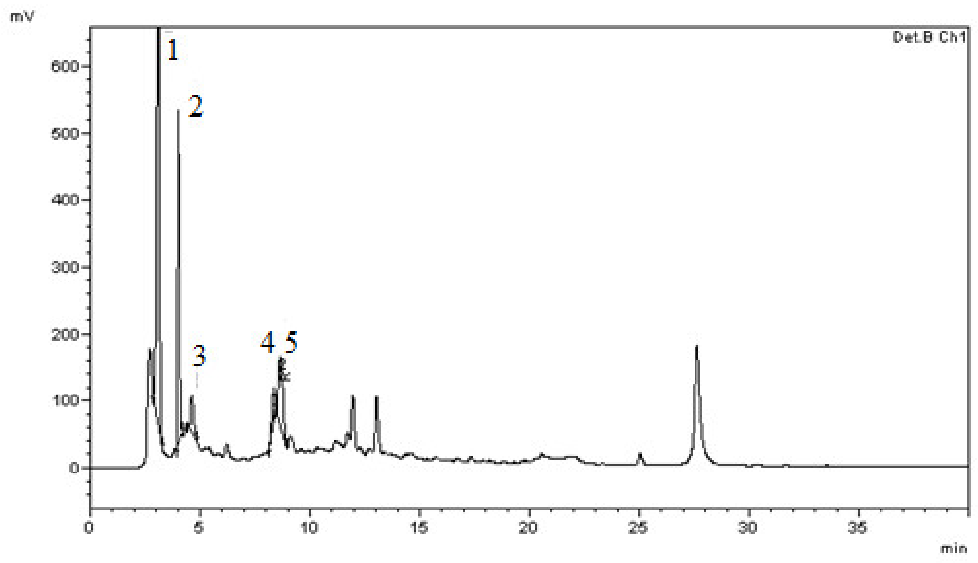

Figure S3. HPLC chromatograms of phenolics in spelt flour (start of storage) on 280 nm: 1 - gallic acid; 2 - protocatechuic acid; 3 - catechin; 4 - epicatechin; 5 - vanillic acid.

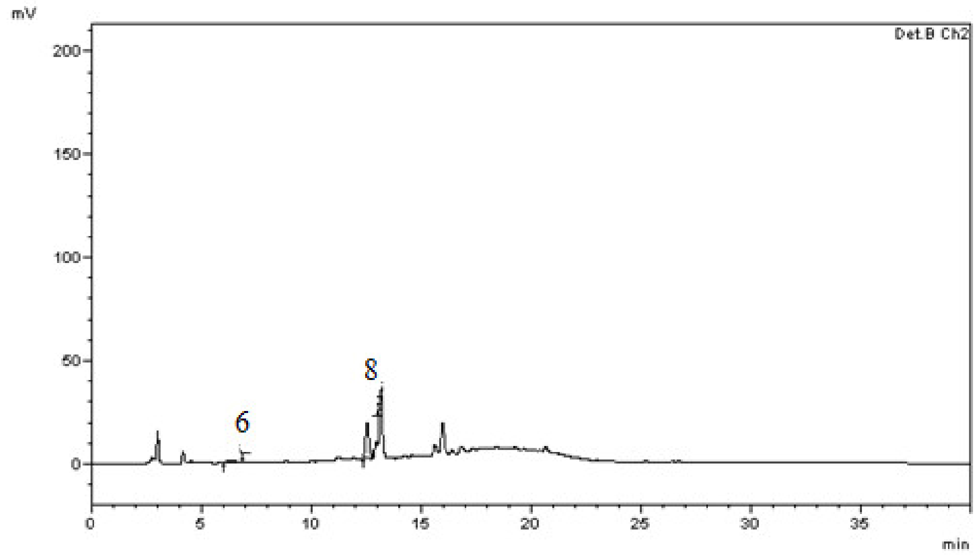

Figure S4. HPLC chromatograms of phenolics in spelt flour (start of storage) on 320 nm: 6 - chlorogeni acid; 7 - *p*-coumaric acid; 8 - caffeic acid.

Table S1. Yeast and mold content in biscuit samples

Sample	Sampling plan		Test results (*n*)-start of storage					Test results (*n*)-3. Months					Test results (*n*)-6. Months				
	n	*c*	1	2	3	4	5	1	2	3	4	5	1	2	3	4	5
**A1**	5	1	< 10	< 10	< 10	< 10	< 10	< 10	< 10	< 10	< 10	< 10	< 10	< 10	< 10	< 10	< 10
**A2**	5	1	< 10	< 10	< 10	< 10	< 10	< 10	< 10	10	< 10	20	10	< 10	< 10	< 10	20
**B1**	5	1	< 10	< 10	< 10	< 10	< 10	< 10	10	< 10	10	< 10	< 10	10	< 10	10	< 10
**B2**	5	1	< 10	< 10	< 10	< 10	< 10	**1600**	**800**	**400**	< 10	**400**	**600**	**1800**	**900**	< 10	**500**
**C1**	5	1	< 10	< 10	< 10	< 10	< 10	< 10	< 10	< 10	< 10	< 10	< 10	< 10	< 10	< 10	< 10
**C2**	5	1	80	< 10	< 10	< 10	< 10	160	< 10	80	< 10	80	100	< 10	< 10	< 10	80
**D1**	5	1	< 10	< 10	< 10	< 10	< 10	< 10	< 10	< 10	< 10	< 10	< 10	< 10	< 10	< 10	< 10
**D2**	5	1	< 10	< 10	< 10	< 10	< 10	< 10	< 10	< 10	< 10	< 10	< 10	< 10	< 10	< 10	< 10

Table S2. Yeast and mold content in spelt flour

Sampling plan		Test results (*n*)-start of storage					Test results (*n*)-3. Months					Test results (*n*)-6. Months				
n	*c*	1	2	3	4	5	1	2	3	4	5	1	2	3	4	5
5	2	50	< 10	< 10	< 10	< 10	**1900**	**1000**	**800**	**600**	**700**	**600**	**800**	**1200**	**600**	**700**

Limit values: *m* – 10 cfu/g; *M* - 10 cfu/g

Table S3. Content of *enterobacteria* in biscuit samples

Sample	Sampling plan		Test results (*n*)-start of storage					Test results (*n*)-3. Months					Test results (*n*)-6. Months				
	n	*c*	1	2	3	4	5	1	2	3	4	5	1	2	3	4	5
**A1**	5	1	< 10	< 10	< 10	< 10	< 10	< 10	< 10	< 10	< 10	< 10	< 10	< 10	< 10	< 10	< 10
**A2**	5	1	< 10	< 10	< 10	< 10	< 10	< 10	< 10	< 10	< 10	< 10	< 10	< 10	< 10	< 10	< 10
**B1**	5	1	< 10	< 10	< 10	< 10	< 10	< 10	< 10	< 10	< 10	< 10	< 10	< 10	< 10	< 10	< 10
**B2**	5	1	< 10	< 10	< 10	< 10	< 10	< 10	< 10	< 10	< 10	< 10	< 10	< 10	< 10	< 10	< 10
**C1**	5	1	< 10	< 10	< 10	< 10	< 10	< 10	< 10	< 10	< 10	< 10	< 10	< 10	< 10	< 10	< 10
**C2**	5	1	< 10	< 10	< 10	< 10	< 10	< 10	< 10	< 10	< 10	< 10	< 10	< 10	< 10	< 10	< 10
**D1**	5	1	< 10	< 10	< 10	< 10	< 10	< 10	< 10	< 10	< 10	< 10	< 10	< 10	< 10	< 10	< 10
**D2**	5	1	< 10	< 10	< 10	< 10	< 10	< 10	< 10	< 10	< 10	< 10	< 10	< 10	< 10	< 10	< 10

Limit values: *m* – 10 cfu/g; *M* - 10 cfu/g

Table S4. Content of aerobic mesophilic bacteria in tea biscuits

Sample	Sampling plan		Test results (*n*)- start of storage	Test results (*n*)- 3. Months	Test results (*n*)- 6. Months
	n	*c*	*n* (1-5)	*n* (1-5)	*n* (1-5)
**A1**	5	5	25	50 (*Bacillus species* 10)	50 (*Bacillus species* 10)
**A2**	5	5	60	100	10
**B1**	5	5	15	20 (*Bacillus cereus* 5)	30
**B2**	5	5	10	40	40
**C1**	5	5	15 (*Bacillus cereus* 10)	20 (*Bacillus cereus* 10)	20
**C2**	5	5	10 (*Bacillus megaterium* 10)	20 (*Bacillus megaterium* 10)	20
**D1**	5	5	30 (*Bacillus megaterium* 20, *Bacillus simplex* 10)	250 (*Bacillus species* 40)	350 (*Bacillus species* 250)
**D2**	5	5	10 (*Bacillus megaterium* 10)	110 (*Bacillus megaterium* 30)	350 (*Bacillus species* 40)
